# Supplementary material for: Molecular structure and interactions within amyloid-like fibrils formed by a low-complexity protein sequence from FUS
Source: Nat Commun. 2020 Nov 12;11:5735. doi: 10.1038/s41467-020-19512-3 (PMC7665218; doi:10.1038/s41467-020-19512-3)
Supplement: Supplementary file 1 — Supplementary Information [file 41467_2020_19512_MOESM1_ESM.pdf]

SUPPLEMENTARY FIGURES FOR:

Molecular Structure and Interactions Within Amyloid-Like Fibrils Formed by a Low-Complexity Protein Sequence from FUS

Myungwoon Lee[1], Ujjayini Ghosh[1], Kent R. Thurber[1], Masato Kato[2,3], and Robert Tycko[1*]

[1]Laboratory of Chemical Physics, National Institute of Diabetes and Digestive and Kidney Diseases, National Institutes of Health, Bethesda, Maryland, USA 20892-0520

[2]Department of Biochemistry, University of Texas Southwestern Medical Center, 5323 Harry Hines Boulevard, Dallas, Texas, USA 75390-9152

[3]Institute for Quantum Life Science, National Institutes for Quantum and Radiological Science and Technology, Chiba, Japan 263-8555.

[*]corresponding author: Dr. Robert Tycko, National Institutes of Health, Building 5, Room 409, Bethesda, MD 20892-0520. e-mail: robertty@mail.nih.gov, phone: 1-301-402-8272, fax: 1-301-496-0825

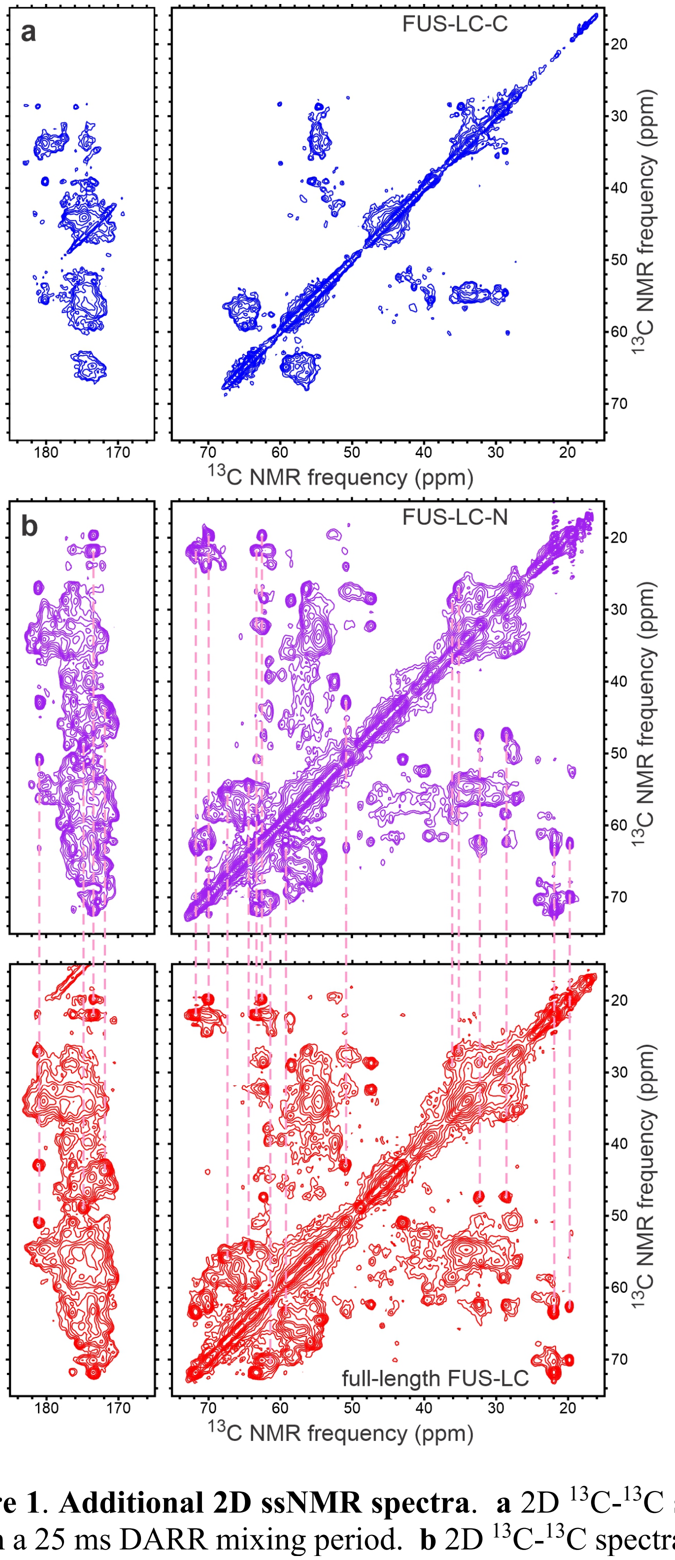

Supplementary Figure 1. **Additional 2D ssNMR spectra**. **a** 2D ^{13}C-^{13}C spectrum of FUS-LC-C fibrils, recorded with a 25 ms DARR mixing period. **b** 2D ^{13}C-^{13}C spectra of FUS-LC-N fibrils (purple) and full-length FUS-LC fibrils (red). Vertical dashed lines, all with the same length, connect corresponding crosspeaks in the two spectra. All fibril samples were uniformly ^{15}N,^{13}C-labeled. Contour levels in all 2D spectra increase by successive factors of 1.5 and were set to show approximately the same number of levels below the maximun signals in all spectra.

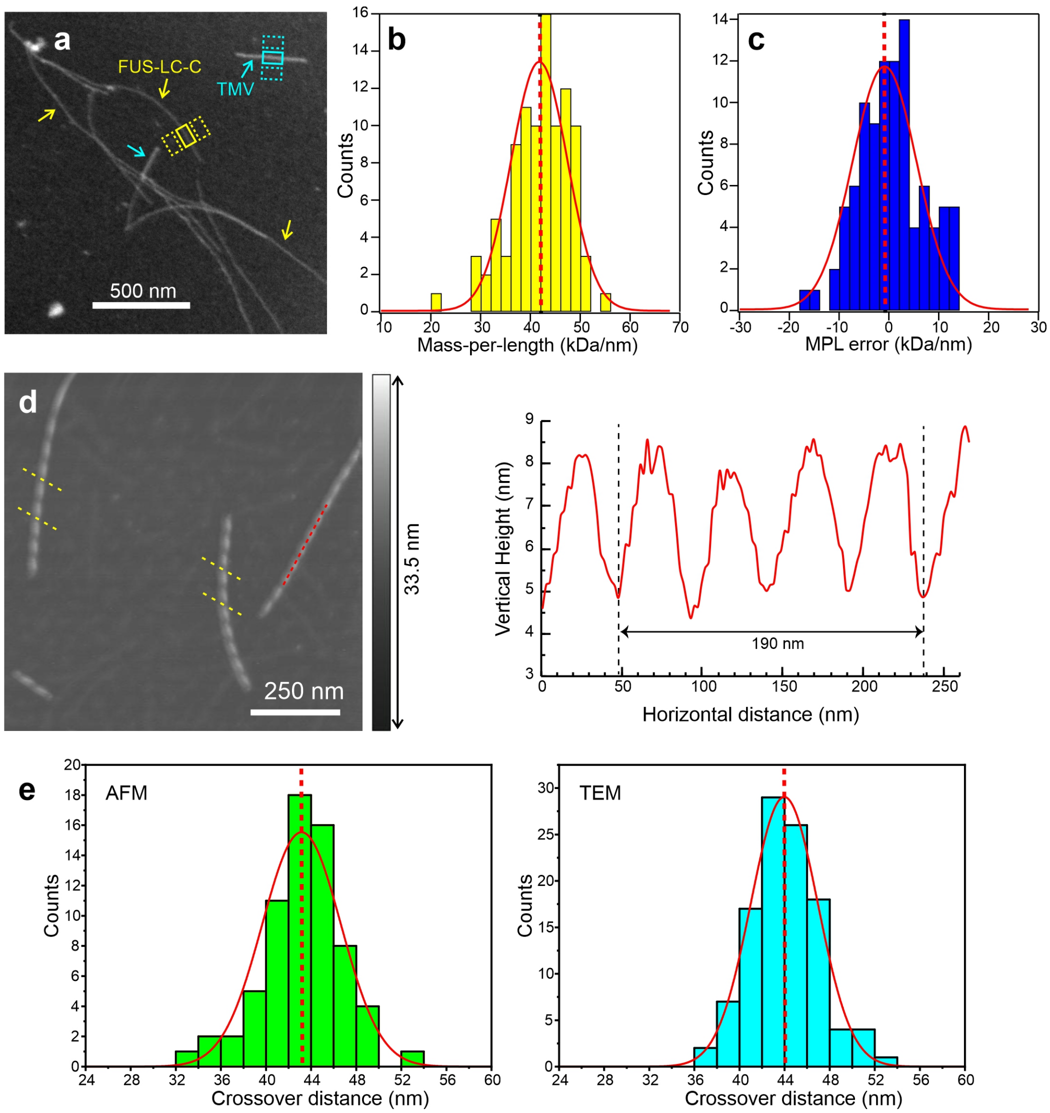

Supplementary Figure 2. **Additional characterization of FUS-LC-C fibrils. a** Example of unstained dark-field TEM image for MPL determination, showing both FUS-LC-C fibrils and TMV rods. Yellow and cyan boxes represent regions used to obtain integrated intensities of fibrils (solid boxes) and background (dashed boxes) for FUS-LC-C fibrils and TMV rods, respectively. **b** Histogram of MPL values determined from 96 fibril segments and 52 TMV segments in seven dark-field TEM images. Red line is a Gaussian fit centered at 41.8 kDa/nm with 8.0 kDa/nm FWHM. **c** Histogram of MPL errors. Red line is a Gaussian fit centered at -1.0 kDa/nm with 9.1 kDa/nm FWHM. **d** AFM height image of FUS-LC-C fibrils. Asymmetry of crossovers, highlighted by yellow dashed lines, indicates a left-handed twist. A height profile along the dashed red line shows minimum and maximum heights of about 5 nm and 8 nm, respectively, in good agreement with Fig. 3a once disordered residues 151-214 are taken into account. **e** Histograms of crossover periods from three AFM images (green bars) and four negative-stain TEM images (cyan bars). Red lines are Gaussian fits centered at 43.2 nm and 44.0 nm, with 8.1 nm FWHM and 7.0 nm FWHM, respectively.

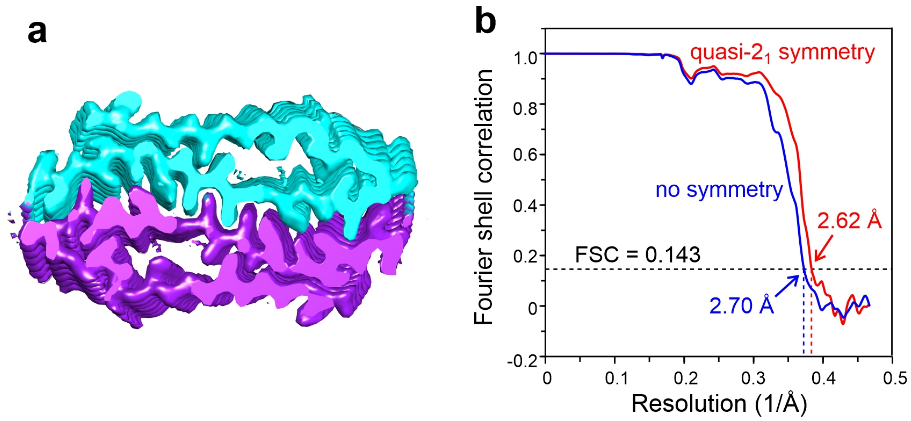

Supplementary Figure 3. **Symmetry and resolution of FUS-LC-C density maps. a** Cryo-EM density map for FUS-LC-C fibrils, calculated without imposing symmetry within the helical repeat unit. Helical rise and twist values are 4.89 Å and -2.11°. **b** Fourier-shell correlation plots for density maps with (blue) and without (red) quasi-2_1 symmetry within the helical repeat unit.

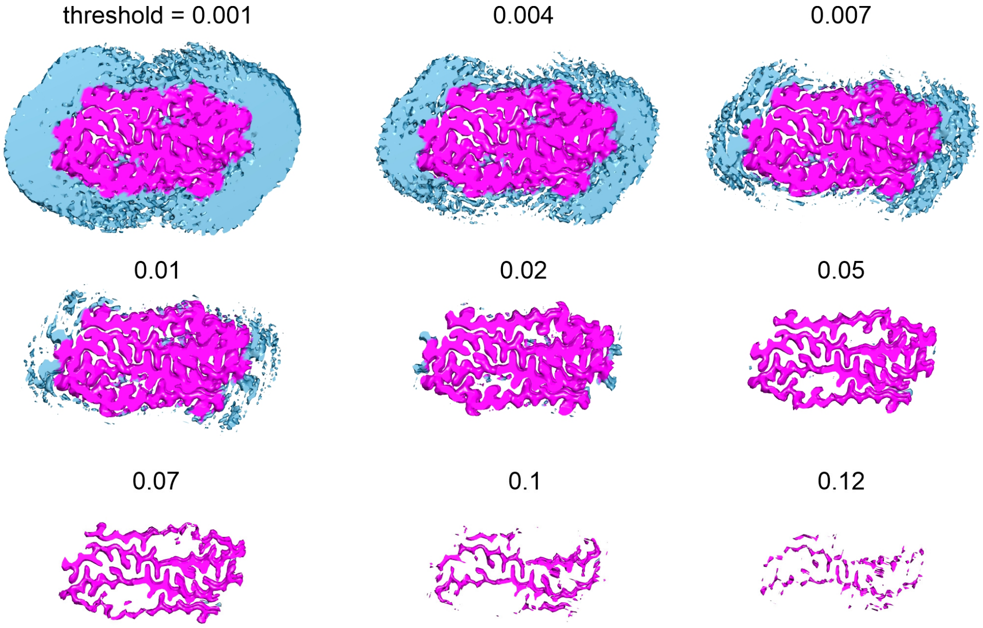

Supplementary Figure 4. Cross-sectional views of the density map for FUS-LC-C fibrils with the indicated threshold values. Threshold values are as reported by Chimera software. The fibril core region and disordered region are colored magenta and blue, respectively. The core region is defined to be density that is within 2.62 Å of atoms in the molecular model shown in Fig. 3a. Magenta and blue regions add up to the full density.

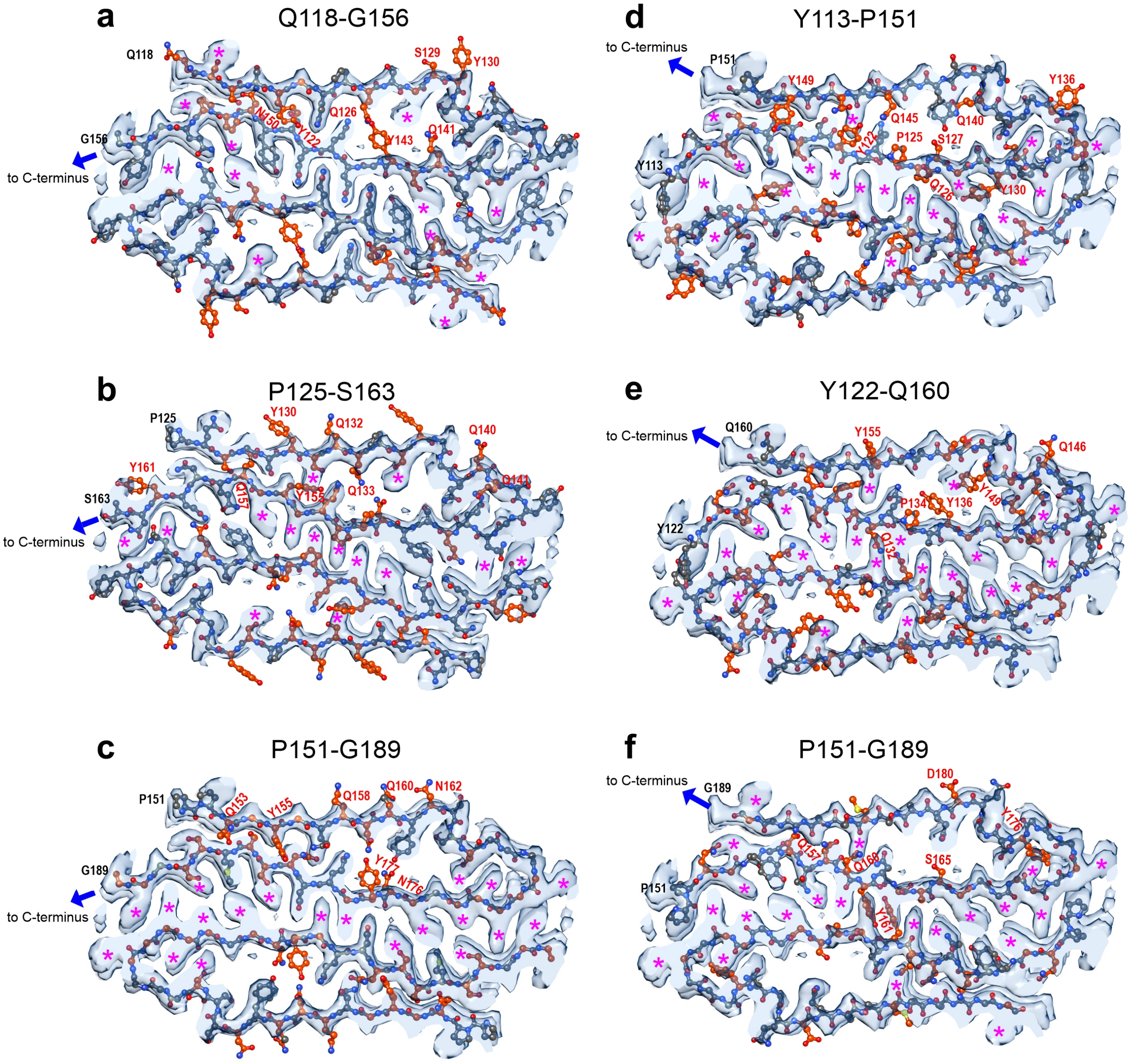

Supplementary Figure 5. **Alternative alignments of the FUS-LC-C sequence in the cryo-EM density map. a,b,c** Attempts to model residues 118-156, 125-163, and 151-189 into the density map, with the N-termini in the outer cross-β density layers and the C-termini in the inner layers. **d,e,f** Attempts to model residues 113-151, 122-160, and 151-189 into density map, with the N-termini in the inner cross-β density layers and C-termini in the outer layers. Carbon atoms of residues that extend outside the density are displayed in orange. Magenta asterisks indicate regions of density that are devoid of protein sidechain atoms.

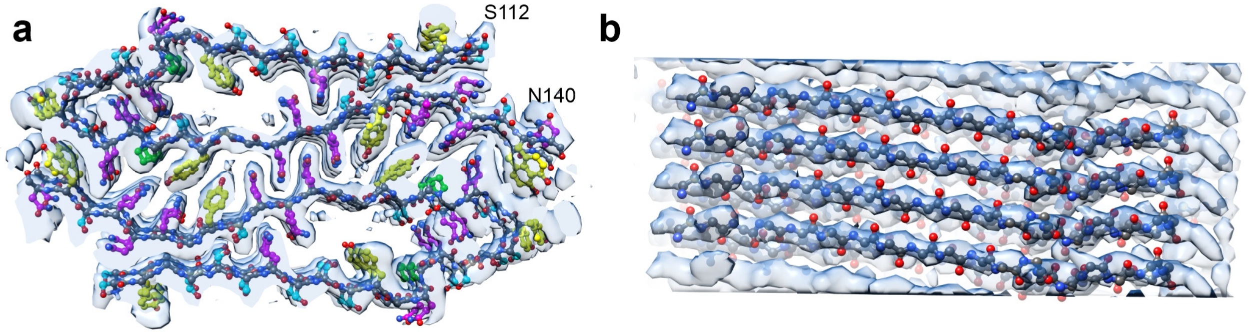

Supplementary Figure 6. **Mirror-image density map and molecular model. a** Cross-sectional view of the mirror-image density map, which has a right-handed twist, and the corresponding molecular model. **b** Side view, showing that the directions of backbone carbonyl groups in the molecular model, which is based on L-amino acids, do not align correctly with backbone corrugations in the mirror-image density map, in contrast to Fig. 3b.

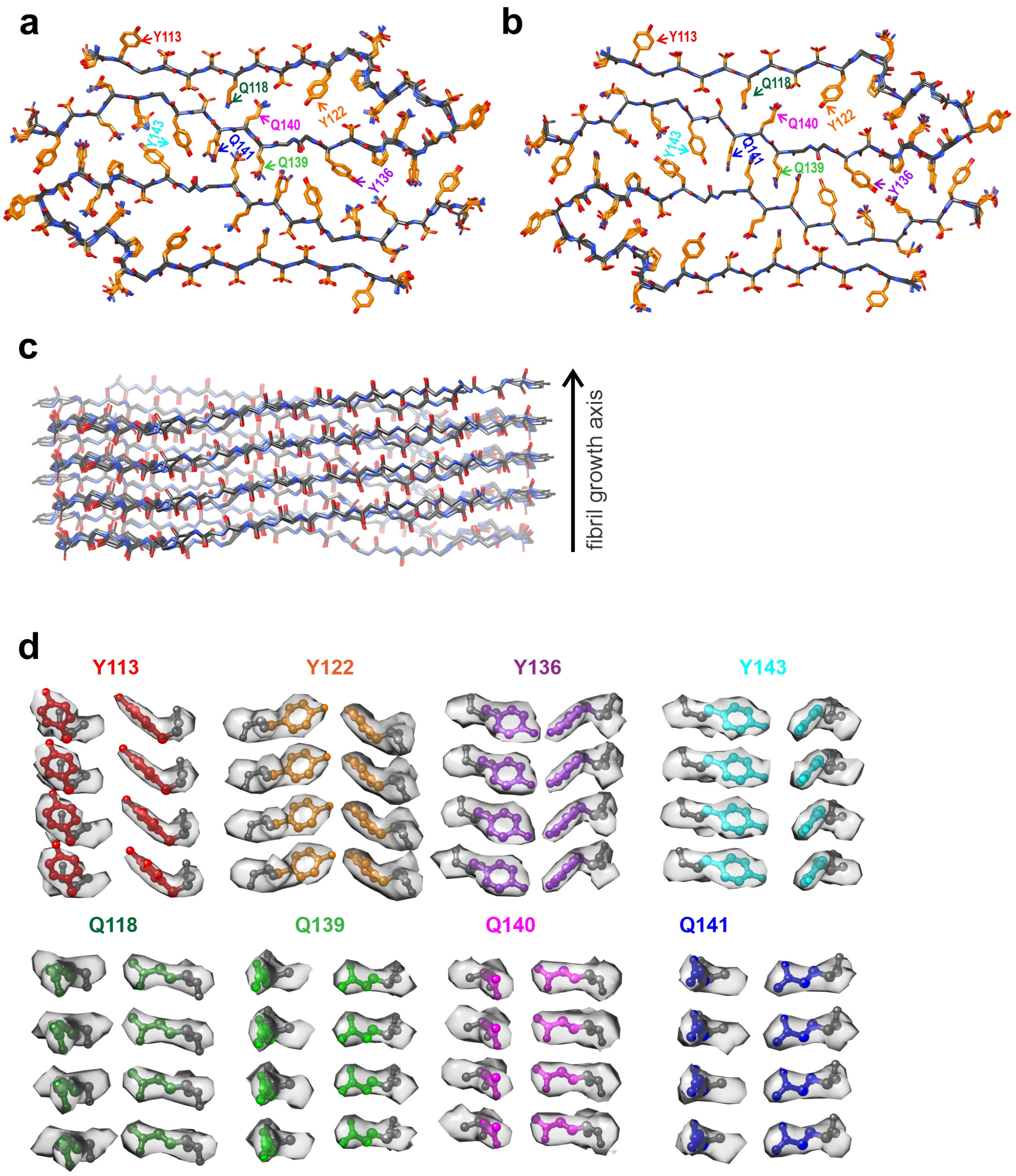

Supplementary Figure 7. Structure bundle from Xplor-NIH calculations. a Cross-sectional view of a superposition of structures from 14 independent calculations in which the cryo-EM density map was used to restrain the positions of all non-hydrogen atoms in residues 112-150. Only one pair of FUS-LC-C molecules from each structure is shown. In these calculations, the TorsionDB potential of Xplor-NIH was used as an additional restraint on sidechain conformation. **b** Same as panel **a**, but a superposition of structures from eight independent calculations in which the torsionDB potential was omitted. **c** Side view of the superposition structures from panel **a**, showing only backbone atoms. **d** Expanded view of density around selected Tyr and Gln residues, color coded to match the labels in panel **a**, illustrating how anisotropies of these densities constrain the sidechain conformations. Each sidechain row (from four copies of FUS-LC, as in panel **c**) is viewed from two directions.

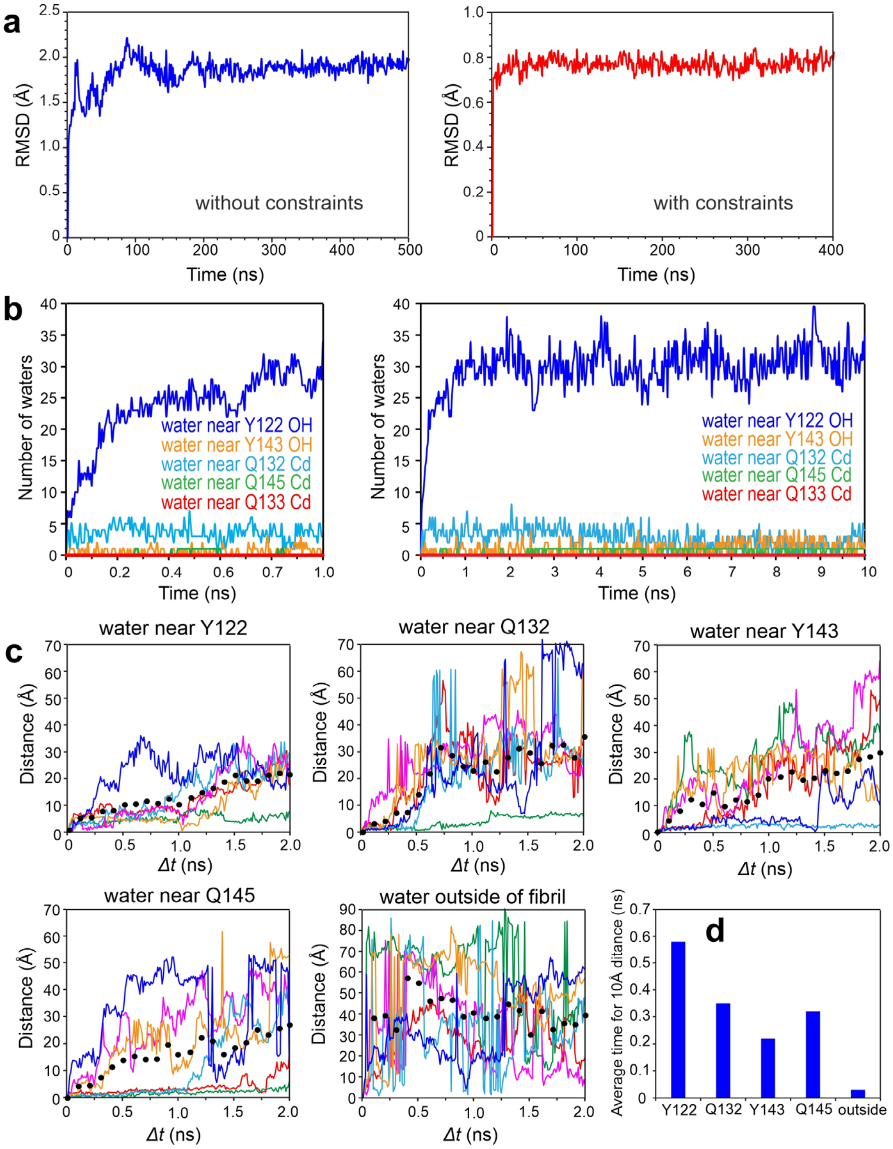

Supplementary Figure 8. Characterization of water within FUS-LC-C fibrils by all-atom MD simulations in explicit water at 303 K. Five repeats of the cryo-EM-based fibril core structure were used, *i.e.*, 10 copies of residues 112-150. No artificial constraints were included. **a** Backbone RMSD of the six central copies, relative to the initial atomic coordinates, in simulations with and without constraints on backbone C_α positions. **b** Time dependences of the number of water molecules within R_{max} of sidechain OH or C_δ sites of the indicated Tyr and Gln residues, for the central pair of FUS-LC-C molecules in the simulated 10-mer. Time zero corresponds to the beginning of the simulation, following energy minimization. For all residues except Q133, R_{max} = 7.0 Å. For Q133, R_{max} = 5.0 Å, chosen because the Q133 sidechain is close to the exterior of the fibril core structure. **c** Movement of water molecules in various locations, quantified as the dependence of the distance from an initial position at time t to the position at t + Δt. Each plot shows results for six water molecules (solid colored lines), with initial positions within 7.0 Å of the sidechain OH or C_δ site of the indicated residue or outside the fibril core. Values of the initial time t are different for different water molecules. Discontinuities in these plots occur when a water molecule crosses a boundary of the periodic simulation box. Black dots are averages over the six water molecules in each plot. **d** Average times for water molecules with the indicated initial positions to move a 10 Å distance (n=6 water molecules for each of the five positions).

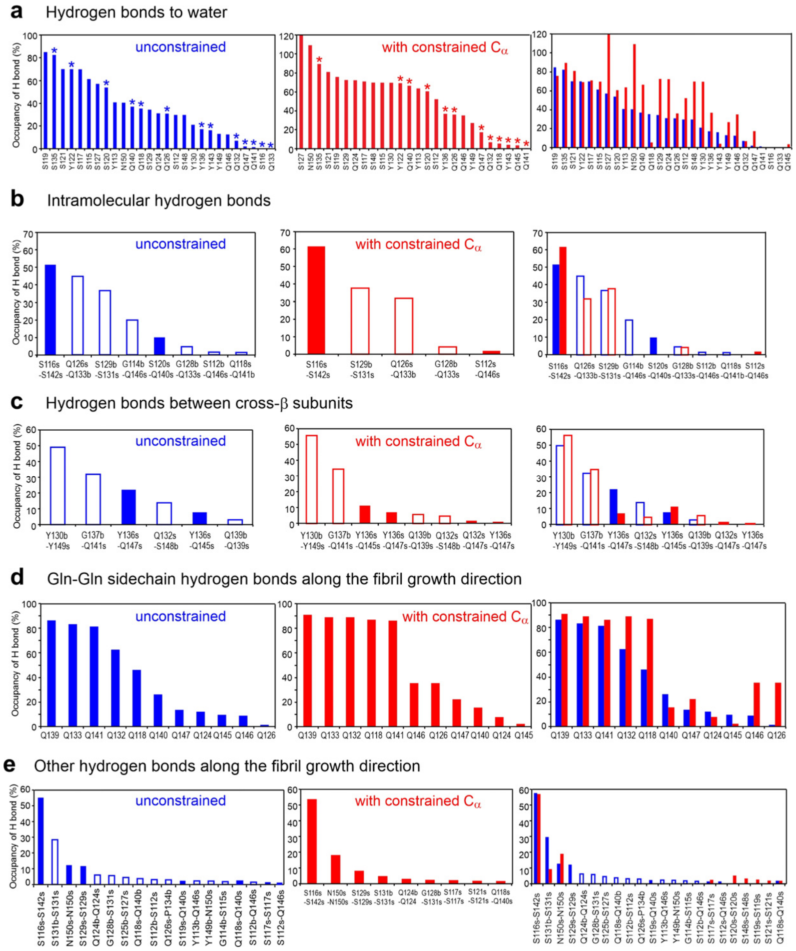

Supplementary Figure 9. Characterization of hydrogen bonds within FUS-LC-C fibrils by all-atom MD in explicit water. A 500 ns trajectory was simulated without constraints. A 400 ns trajectory was simulated with position constraints on backbone Cα sites. **a** Occupancies of hydrogen bonds between water and sidechains of the indicated residues, averaged over the unconstrained (blue) and constrained (red) trajectories. Asterisks indicate sidechains that are not solvent-exposed. Values greater than 100% arise from simultaneous participation of a given sidechain in hydrogen bonds to two different water molecules. Occupancies are averages of results for the two central FUS-LC-C molecules in the simulated 10-mer. **b** Occupancies of intramolecular hydrogen bonds between amino acid sidechains (solid bars, s-s) and between the sidechain of one residue and the backbone of another residue (open bars, b-s or s-b), averaged over the unconstrained (blue) and constrained (red) trajectories. Occupancies are averages of results for the two central FUS-LC-C molecules in the simulated 10-mer. **c** Occupancies of hydrogen bonds between the two cross-β subunits of the FUS-LC-C fibril core, including sidechain-sidechain hydrogen bonds (solid bars, s-s) and sidechain-backbone hydrogen bonds (open bars, b-s or s-b), averaged over the unconstrained (blue) and constrained (red) trajectories. Occupancies are averages of results for the central FUS-LC-C molecule in each subunit, interacting with molecules in the opposite subunit. **d** Occupancies of intermolecular Gln-Gln sidechain hydrogen bonds along the fibril growth direction (*i.e.*, polar zipper interactions), averaged over the unconstrained (blue) and constrained (red) trajectories. Occupancies are averages of results for the central FUS-LC-C molecule in each cross-β subunit, interacting with a nearest-neighbor molecule in the same subunit (n=2 molecules). **e** Occupancies of other intermolecular sidechain-sidechain (solid bars, s-s) and sidechain-backbone (open bars, b-s or s-b) hydrogen bonds along the fibril growth direction, averaged over the unconstrained (blue) and constrained (red) trajectories. Occupancies are averages of results for the central FUS-LC-C molecule in each cross-β subunit (n=2 molecules), interacting with a nearest-neighbor molecule in the same subunit.

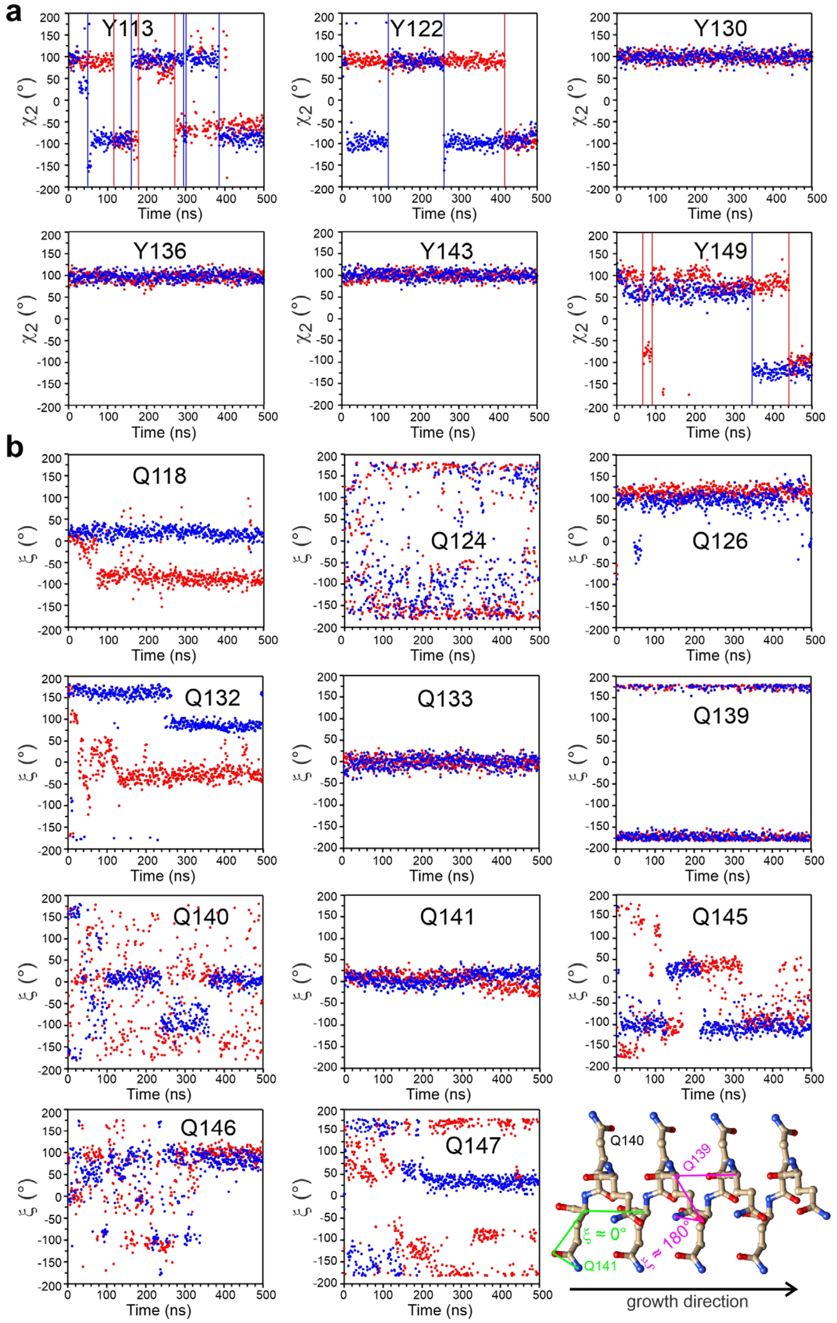

Supplementary Figure 10. Sidechain conformational dynamics in FUS-LC-C fibrils, from a 500 ns MD trajectory without constraints. Results are shown for the central molecules in each of the two cross-β subunits of the simulated 10-mer (red and blue). **a** Time dependences of sidechain χ_2 torsion angles for Tyr residues. Discontinuities in χ_2 (red and blue vertical lines) indicate Tyr ring flips. **b** Time dependences of the pseudo-torsion angle ξ for Gln residues, defined by the four atoms $N_{\varepsilon 2}$(i)-$O_{\varepsilon 1}$(i)-C_α(i)-C_α(i+1). As shown in the example for Q139 (magenta labels) and Q141 (green labels), values of ξ near 0° or ±180° correspond to parallel or antiparallel alignments of the Gln sidechain amide group relative to the fibril growth direction, *i.e.*, opposite directions for Gln-Gln polar zipper interactions.

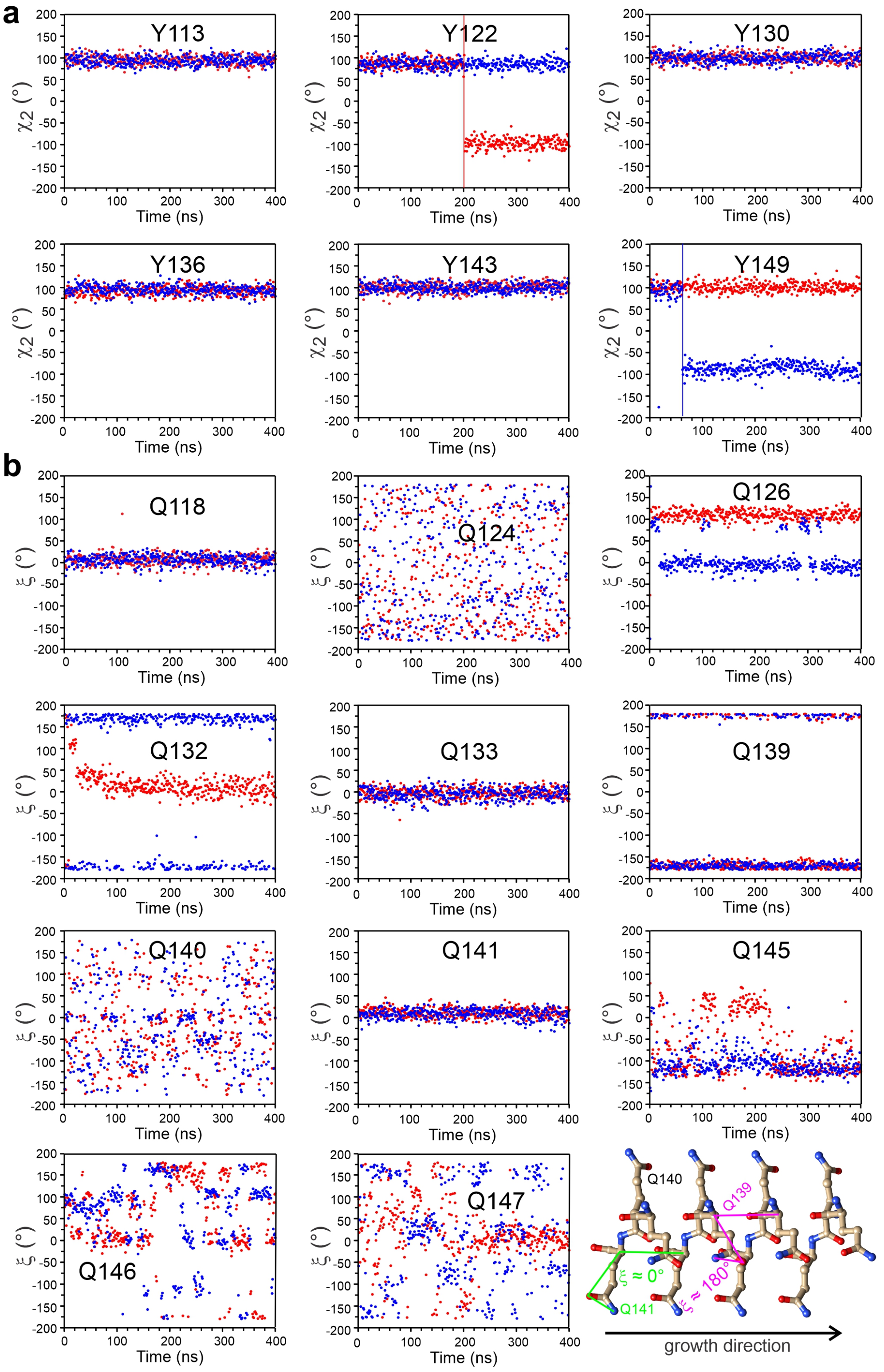

Supplementary Figure 11. Same as Supplementary Fig. 10, but from a 400 ns MD trajectory with position constraints on backbone C_α sites.

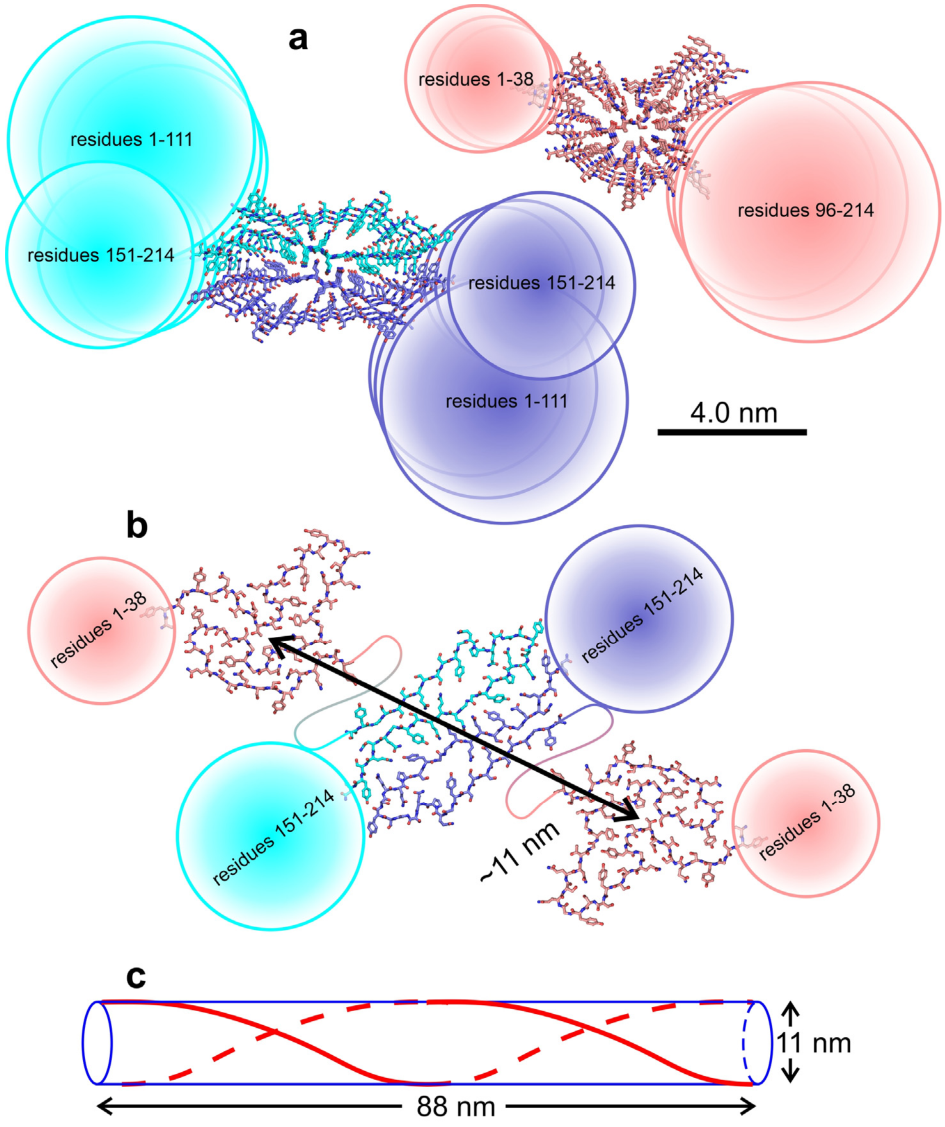

Supplementary Figure 12. **Factors proposed to affect FUS-LC fibril structure.** **a** Schematic illustration of volumes occupied by disordered segments of full-length FUS-LC fibrils with the experimentally observed cross-β core formed by residues 39-95 (pink, PDB code 5W3N, http://doi.org/10.2210/pdb5W3N/pdb), or by disordered segments of hypothetical full-length FUS-LC fibrils with a cross-β core formed by residues 112-150 (blue and cyan, PDB code 6XFM, http://doi.org/10.2210/pdb6XFM/pdb). Disordered segments are represented by spheres with radii equal to 3.14 Å × $N^{0.5}$, where N is the number of residues, corresponding approximately to experimentally determined radii of gyration of intrinsically disordered proteins[16]. Fibril structures are viewed in cross-section. **b** Cross-section of a hypothetical full-length FUS-LC fibril structure containing a 2_1-symmetric central core formed by residues 112-150 and peripheral cores formed by residues 39-95. The centers of the two peripheral cores are separated by about 11 nm. **c** Spiral paths (red lines) followed by the centers of the peripheral cores as the hypothetical fibril structure in panel **b** twists about the fibril growth axis. The longer path length for peripheral cores, relative to the central core, would require stretching of intermolecular distances by nearly 0.4 Å on average.
