# Supplementary material for: Molecular structure and interactions within amyloid-like fibrils formed by a low-complexity protein sequence from FUS
Source: Nat Commun. 2020 Nov 12;11:5735. doi: 10.1038/s41467-020-19512-3 (PMC7665218; doi:10.1038/s41467-020-19512-3)
Supplement: Supplementary file 3 — Description of Additional Supplementary Files [file 41467_2020_19512_MOESM3_ESM.pdf]

**Description of Additional Supplementary Files**

File Name: Supplementary Movie 1
Description: MD simulation of the FUS-LC-C fibril core in 100 mM NaCl at 303 K, with constraints on positions of $C_\alpha$ atoms.

File Name: Supplementary Movie 2
Description: MD simulation of the FUS-LC-C fibril core in 100 mM NaCl at 303 K, without constraints. Apparent movements of the FUS-LC-C assembly relative to solvent molecules arise from diffusion across periodic boundaries and does not indicate real desolvation.
