# Supplementary material for: Molecular structure and interactions within amyloid-like fibrils formed by a low-complexity protein sequence from FUS
Source: Nat Commun. 2020 Nov 12;11:5735. doi: 10.1038/s41467-020-19512-3 (PMC7665218; doi:10.1038/s41467-020-19512-3)
Supplement: Supplementary file 6 — Reporting Summary [file 41467_2020_19512_MOESM6_ESM.pdf]

nature research

Corresponding author(s): Robert Tycko

Last updated by author(s): 10/1/2020

Reporting Summary

Nature Research wishes to improve the reproducibility of the work that we publish. This form provides structure for consistency and transparency in reporting. For further information on Nature Research policies, see our Editorial Policies and the Editorial Policy Checklist.

Statistics

For all statistical analyses, confirm that the following items are present in the figure legend, table legend, main text, or Methods section.

n/a	Confirmed	
☐	☒	The exact sample size (*n*) for each experimental group/condition, given as a discrete number and unit of measurement
☐	☒	A statement on whether measurements were taken from distinct samples or whether the same sample was measured repeatedly
☒	☐	The statistical test(s) used AND whether they are one- or two-sided<br>*Only common tests should be described solely by name; describe more complex techniques in the Methods section.*
☒	☐	A description of all covariates tested
☒	☐	A description of any assumptions or corrections, such as tests of normality and adjustment for multiple comparisons
☐	☒	A full description of the statistical parameters including central tendency (e.g. means) or other basic estimates (e.g. regression coefficient) AND variation (e.g. standard deviation) or associated estimates of uncertainty (e.g. confidence intervals)
☒	☐	For null hypothesis testing, the test statistic (e.g. *F*, *t*, *r*) with confidence intervals, effect sizes, degrees of freedom and *P* value noted<br>*Give P values as exact values whenever suitable.*
☒	☐	For Bayesian analysis, information on the choice of priors and Markov chain Monte Carlo settings
☒	☐	For hierarchical and complex designs, identification of the appropriate level for tests and full reporting of outcomes
☒	☐	Estimates of effect sizes (e.g. Cohen's *d*, Pearson's *r*), indicating how they were calculated

Our web collection on statistics for biologists contains articles on many of the points above.

Software and code

Policy information about availability of computer code

Data collection	CryoEM data, dark-field TEM data, and NMR data were collected with SerialEM 3.7, AMT Image Capture Engine 6.02, and Spinsight 4.3.2, respectively.
Data analysis	CryoEM data were processed with RELION 3.0. Molecular models were developed with Coot 0.8.9.2, Chimera 1.14, and Xplor-NIH 2.53. MD simulations were performed with NAMD 2.12 and CHARMM22 potentials, and and analyzed with VMD 1.9.3. Dark-field TEM images were analyzed with ImageJ 1.52 and Igor Pro 7. NMR data were analyzed with nmrPipe 9.4 and Sparky 3.114. MatLab scripts used in cryoEM data processing are available upon request.

For manuscripts utilizing custom algorithms or software that are central to the research but not yet described in published literature, software must be made available to editors and reviewers. We strongly encourage code deposition in a community repository (e.g. GitHub). See the Nature Research guidelines for submitting code & software for further information.

Data

Policy information about availability of data

All manuscripts must include a data availability statement. This statement should provide the following information, where applicable:
- Accession codes, unique identifiers, or web links for publicly available datasets
- A list of figures that have associated raw data
- A description of any restrictions on data availability

The cryo-EM density map for FUS-LC-C fibrils is available from the Electron Microscopy Data Bank, code EMD-22169. Atomic coordinates for a bundle of 14 molecular structures that fit the density map are available from the Protein Data Bank, code 6XFM. 2D ssNMR spectra are available at http://dx.doi.org/10.17632/ts9p355m3d.2. All other data are available from the authors upon request.

Field-specific reporting

Please select the one below that is the best fit for your research. If you are not sure, read the appropriate sections before making your selection.

- [x] Life sciences
- [ ] Behavioural & social sciences
- [ ] Ecological, evolutionary & environmental sciences

For a reference copy of the document with all sections, see nature.com/documents/nr-reporting-summary-flat.pdf

Life sciences study design

All studies must disclose on these points even when the disclosure is negative.

Sample size	The final cryo-EM density map results from 275,520 particles (i.e., overlapping fibril segments), which were extracted from 2411 images.
Data exclusions	In the cryo-EM image analysis process, particles were grouped by the software into multiple 2D classes. 2D classes that did not contribute to a high-resolution 3D density map were discarded, resulting in final retention of 55% of the original image particles (see Table 1).
Replication	Replication was not performed explicitly to test reproducibility, but identical FUS-LC-C fibril morphologies were obtained in five independent sample preparations.
Randomization	Randomization was used only for the Fourier-shell correlation analysis in Supplementary Fig. 3b. Randomization serves no purpose in other analyses.
Blinding	Blinding was not used because this is a study of molecular structure and dynamics. No subjective assessments of groups or categories were involved. Blinding would serve no purpose.

Reporting for specific materials, systems and methods

We require information from authors about some types of materials, experimental systems and methods used in many studies. Here, indicate whether each material, system or method listed is relevant to your study. If you are not sure if a list item applies to your research, read the appropriate section before selecting a response.

Materials & experimental systems

n/a	Involved in the study	
[x]	[ ]	Antibodies
[x]	[ ]	Eukaryotic cell lines
[x]	[ ]	Palaeontology and archaeology
[x]	[ ]	Animals and other organisms
[x]	[ ]	Human research participants
[x]	[ ]	Clinical data
[x]	[ ]	Dual use research of concern

Methods

n/a	Involved in the study	
[x]	[ ]	ChIP-seq
[x]	[ ]	Flow cytometry
[x]	[ ]	MRI-based neuroimaging
